# Rejuvenation of Meropenem by Conjugation with Tilapia Piscidin-4 Peptide Targeting NDM-1 *Escherichia coli*

**DOI:** 10.1021/acsomega.4c03352

**Published:** 2024-06-28

**Authors:** Sanjay Prasad Selvaraj, Kuan-Hung Lin, Wen-Chun Lin, Ming-Feng You, Tsung-Lin Li, Jyh-Yih Chen

**Affiliations:** † Molecular and Biological Agricultural Science Program, Taiwan International Graduate Program, Academia Sinica, Taipei 11529, Taiwan; ‡ Graduate Institute of Biotechnology, National Chung Hsing University, Taichung 402, Taiwan; § Genomics Research Center, Academia Sinica, Taipei 11529, Taiwan; ∥ Marine Research Station, Institute of Cellular and Organismic Biology, Academia Sinica, 23-10 Dahuen Rd, Jiaushi, Ilan 262, Taiwan; ⊥ Biotechnology Center, National Chung Hsing University, Taichung City 402, Taiwan; # The iEGG and Animal Biotechnology Center and the Rong Hsing Research Center for Translational Medicine, National Chung Hsing University, Taichung 402, Taiwan

## Abstract

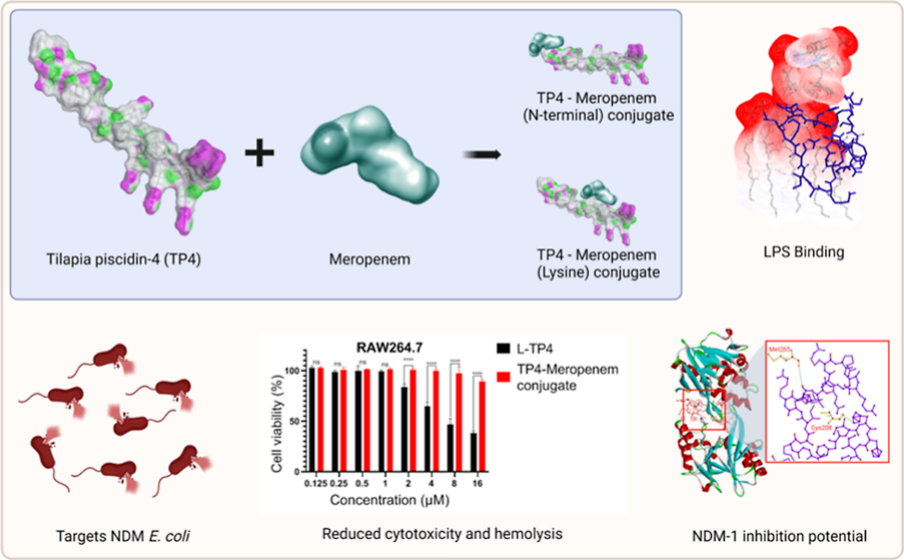

Gram-negative pathogens that produce β-lactamases pose a serious public health threat as they can render β-lactam antibiotics inactive via hydrolysis. This action contributes to the waning effectiveness of clinical antibiotics and creates an urgent need for new antimicrobials. Antimicrobial peptides (AMPs) exhibiting multimodal functions serve as a potential source in spite of a few limitations. Thus, the conjugation of conventional antibiotics with AMPs may be an effective strategy to leverage the advantages of each component. In this study, we conjugated meropenem to the AMP Tilapia piscidin 4 (TP4) using a typical coupling reaction. The conjugate was characterized by using HPLC-MS, HR-MS, and MS–MS fragmentation analysis. It was then evaluated in terms of antibacterial potency, hemolysis, and cytotoxicity toward RAW264.7 and CCD-966SK cell lines. The conjugation of meropenem with TP4 significantly reduced the cytotoxicity compared to TP4. Conjugation of unprotected TP4 with meropenem resulted in cross-linking at the N-terminal and lysine sites. The structural activity relationship of the two isomers of the TP4-meropenem conjugate was investigated. Both the isomers showed notable antibacterial activities against NDM-1 *Escherichia coli* and reduced red blood cell hemolysis as compared to TP4. Lysine conjugate (TP4-K-Mero) showed lesser hemolysis than the N-terminal conjugate (TP4-N-Mero). Molecular modeling further revealed that the conjugates can bind to lipopolysaccharides and inhibit NDM-1 β-lactamase. Together, these data show that conjugation of antibiotics with AMP can be a feasible approach to increase the therapeutic profile and effectively target multidrug-resistant pathogens. Furthermore, antibiotic conjugation at different AMP sites tends to show unique biological properties.

## Introduction

The emergence of drug-resistant pathogens has been largely attributed to abnormal and improper use of antibiotics in agriculture and healthcare settings.^1,2^ Antimicrobial-resistant (AMR) pathogens are considered to be among the most urgent threats to public health by the World Health Organization (WHO), as a substantial rise in the death rate due to AMR infections has been seen in recent decades. According to a recent UN report, around 700,000 people die each year because of AMR infections, with 230,000 succumbing to multidrug-resistant (MDR) tuberculosis infections.^3^

In 2019, the WHO and the US Centers for Disease Control and Prevention highlighted carbapenem-resistant pathogens as the most dangerous microbial threat.^4^ In these organisms, resistance is conferred by one of several β-lactam hydrolyzing enzymes, collectively termed carbapenamases (β-lactamases). These enzymes break down critical antibiotics including carbapenems, penicillins, cephalosporins, and monobactams. The carbapenemase enzymes are classified into four Ambler classes: A, B, C, and D.^5^ Among the classes, some of the most concerning carbapenemases include: the New Delhi metallo-β-lactamase 1 (NDM-1) and *K. pneumoniae* carbapenemase (KPC) from class A; Verona integron-encoded metallo-β-lactamase (VIM) and imipenem-resistant *pseudomonas* (IMP) from class B; and oxacillinase-48 (OXA-48) from class D. Carbapenems such as meropenem, imipenem, doripenem, and ertapenem are utilized as last-resort antibiotics for treating lethal infections caused by MDR bacteria. However, horizontal gene transfer and spread of carbapenemase genes have rendered carbapenems largely ineffective in many cases and led to the emergence of highly virulent strains with high mortality.^6,7^

Membrane-active antimicrobial peptides (AMPs) have been lauded as potential candidates for the development of next-generation antimicrobials. Unlike classical antibiotics, AMPs target the bacterial cell membranes in their primary modes of action. The amphipathic property of AMPs enhances membrane penetration and causes a disruption of cell membrane integrity. The initial association with negatively charged bacterial cell membranes is due to cationic amino acids, while hydrophobic amino acids facilitate subsequent membrane penetration. A few AMPs are internalized into the cell and target internal organelles. Since membrane disruption and organelle targeting both lead to rapid killing of the cell, there is a relatively lower chance that microbes will develop resistance to AMPs than to conventional antibiotics.^8−10^ Tilapia piscidin-4 (TP4) is a cationic AMP consisting of 25 amino acids (FIHHIIGGLFSAGKAIHRLIRRRRR) that has a broad range of potential therapeutic functions. For example, TP4 exhibits antimicrobial, anticancer, immunomodulatory, and wound-healing activities. It contains a net positive charge of +10, largely due to a pentaarginine cluster at the C-terminus. There is a single internal lysine residue, which contributes to the propensity for TP4 to adopt an α-helical secondary structure in solution. Most importantly, TP4 is able to inhibit MDR strains and eradicate biofilms due to its capability to form pores in the membranes of pathogens.^11−13^

Despite these notable advantages, AMPs have some key limitations, such as sensitivity to degradation by gastric, intestinal, and serum proteases, nonspecific toxicity, and reduced activity at physiological pH and salt concentrations.^14,15^ Structural modifications may be introduced into the peptides in order to overcome these drawbacks and increase their therapeutic efficacy. For instance, amino acid substitutions, cyclization, stapling, conjugation, and peptidomimetics are strategies that have been widely applied to improve the potential of AMPs as pharmacological agents. Among these strategies, conjugation with conventional antibiotics has become of special interest, as AMP-antibiotic hybrids often show enhanced efficacy against AMR pathogens concomitant with other desirable biological properties.^16^

Conjugation of AMPs with antibiotics is a potential technique to leverage the advantages and overcome the limitations of individual component molecules. When two functional molecules are conjugated, a synergistic effect may occur, with the conjugate outperforming either individual constituent.^16^ Therefore, the conjugation strategy is considered a useful means of rejuvenating or reutilizing antibiotics for AMR pathogens.^17,18^

In this study, we chemically conjugated TP4 with the β-lactam antibiotic Meropenem in an effort to overcome Meropenem resistance in pathogens. Meropenem was chosen for cross-linking as it belongs to the carbapenem class of antibiotics, which are considered one of the last-resort antibiotics for the treatment of fatal MDR infections, and resistance to Meropenem has been widely reported in recent years.^19^ The resulting conjugates were evaluated in terms of antibacterial activity against drug-sensitive and MDR strains. The toxicity of the conjugates was further evaluated by the alamarBlue assay on RAW murine macrophage cells and CCD966-SK human fibroblast cells; hemolysis was also assessed using sheep red blood cells (RBCs). Molecular docking analysis was then performed to explore the interactions of the conjugates with lipopolysaccharides (LPS) as well as their potential binding to NDM-1 β-lactamase. The potential for penetration of Gram-negative cell membranes was analyzed *in silico* using the PPM server.

## Results and Discussion

### Synthesis of TP4-Meropenem Conjugates

**Scheme 1:**
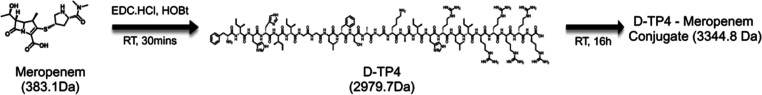
Synthesis Scheme of D-TP4-Meropenem Conjugates The carboxyl group of meropenem was activated using *N*-ethyl-*N*′-(3-dimethylaminopropyl) carbodiimide hydrochloride (EDC.HCl) and hydroxybenzotriazole (HOBt). Subsequently, activated meropenem reacted with TP4, resulting in formation of AMP-antibiotic conjugates.

**Figure 1:**
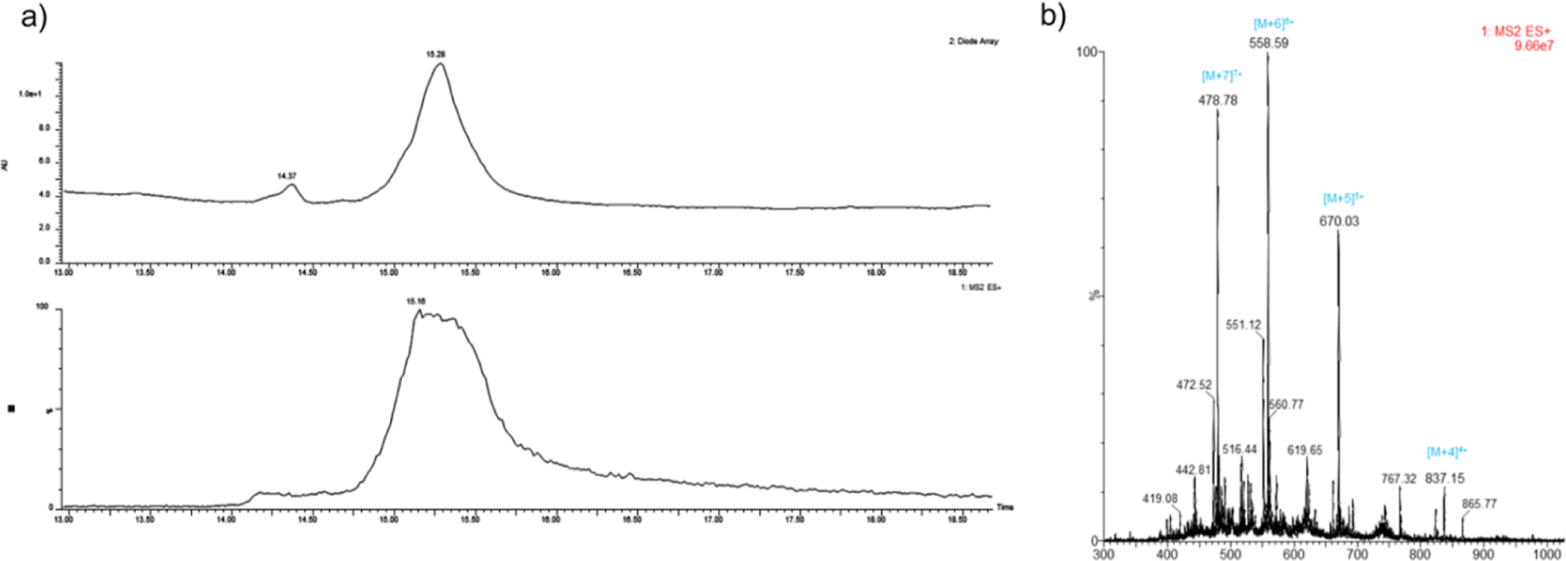
Characterization of the D-TP4-meropenem conjugate. (a) HPLC-MS chromatogram; (b) mass spectrum showing the *m*/*z* values corresponding to the calculated molecular weight.

**Figure 2:**
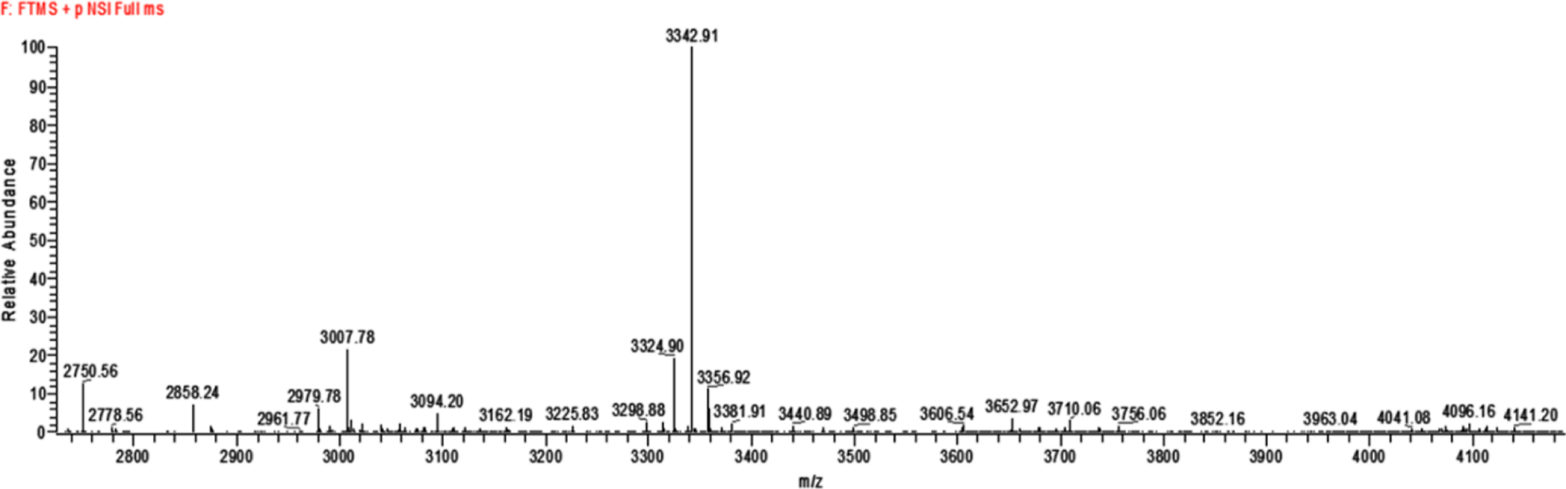
HR-MS analysis of the D-TP4-Meropenem conjugate.

The conjugation of D-Tilapia piscidin 4 (D-TP4) with meropenem was synthesized using carbodiimide reaction chemistry, wherein amide bonds are formed between the carboxyl and amine groups of meropenem and TP4. The carboxyl group of Meropenem was activated prior to the addition of TP4 (Scheme 1). The resulting D-TP4-meropenem conjugates were purified and analyzed using high-performance liquid chromatography-mass spectrometry (HPLC-MS) and high-resolution-mass spectrometry (HR-MS). The conjugates were obtained with high purity, as shown in the HPLC-MS chromatogram (Figure 1a). The MS spectrum with charge states is shown in Figure 1b; *m*/*z* values of 837.14 [M + 4]^+4^, 670.03 [M + 5]^+5^, 558.59 [M + 6]^+6^, and 478.78 [M + 7]^+7^ all are convergent to a calculated molecular weight of 3344.8 Da after deconvolution (Figure 1b). The observed molecular weight of the conjugates was further confirmed by HR-MS analysis, which yielded a value of 3342.91 g/mol (Figure 2), indicating successful synthesis and purification of the D-TP4-meropenem conjugate (purity >95%; 96.32%). In order to confer resistance to proteases such as trypsin, chymotrypsin, proteinase K, and pepsin, TP4 composed of d-amino acids was utilized for conjugation in this study. l-Amino acids form right-handed helices while d-amino acids form left-handed helices; thus, altering the chirality of AMPs can prevent recognition by proteases.^20,21^

### Antibacterial Activity of Conjugates

The synthesized conjugates were examined for their antibacterial activity against selected Gram-positive and Gram-negative clinical strains. L-TP4, D-TP4, and meropenem were used as controls to evaluate the comparative antimicrobial effectiveness. The TP4-meropenem conjugate showed a similar activity profile to L-TP4 and D-TP4 when tested against Gram-negative *Escherichia coli* and *Acinetobacter baumannii* (Table 1).

**Table 1:** Antibacterial Activity of D-TP4-meropenem Conjugate^a^

AMP/conjugate	minimum inhibitory concentration (MIC)		
	*E. coli* 8–8	*A. baumannii* 2982	*S. aureus* 13–1
L-TP4	2–4	2–4	4–8
D-TP4	2–4	2–4	2–4
D-TP4-Meropenem conjugate	4	4	32
Meropenem	<0.25	>32	0.25

a Values are expressed in (μM).

### Cytotoxicity Analysis of Conjugates

**Figure 3:**
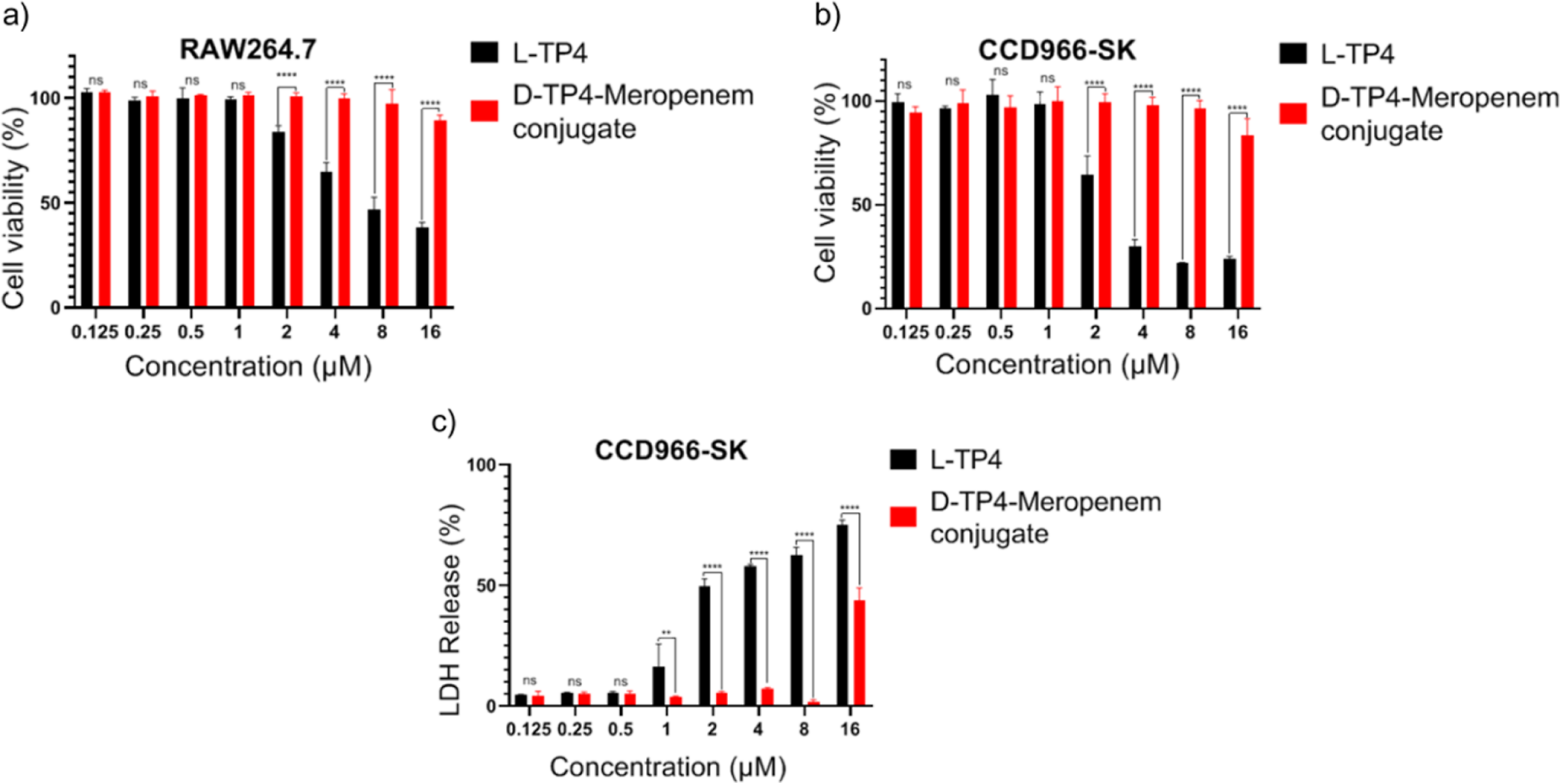
Cytotoxicity analysis of the conjugates. Cytotoxicity analysis on RAW264.7 (a) and CCD966-SK cells, (b) by alamarBlue assay, and (c) LDH leakage assay on CCD966-SK cells. Significant differences compared to the control were identified by two-way ANOVA and Tukey’s multiple comparison test (*p < 0.05; **p < 0.01; ***p < 0.001; ****p < 0.0001, ns—no statistical significance).

The conjugates were then assayed for cytotoxicity toward murine macrophages (RAW264.7) and human fibroblasts (CCD966-SK) using the alamarBlue assay. The TP4-meropenem conjugate showed significantly lower toxicity than TP4 for both cell lines (Figure 3a,b). From the cytotoxicity analysis of L-TP4 and D-TP4, D-TP4 showed a slightly higher toxicity than L-TP4 (Supporting Information Figure S1). Treatment of RAW264.7 and CCD966-SK cells with 16 μM TP4 led to cell viabilities of 38.2 ± 2.5% and 23.9 ± 1.2%, whereas 16 μM conjugates led to viabilities of 89.4 ± 2.5% and 83.5 ± 8.0%. The LDH leakage assay showed drastically less release of LDH when cells were treated with conjugates compared with TP4 (Figure 3c). These results suggest that conjugation of Meropenem with TP4 improves the cytotoxicity profile of the toxic AMP.

Nonspecific toxicity to host cells is a major concern in the potential application of AMP therapeutics. The natural form of TP4 features evident cytotoxicity and hemolysis, making its therapeutic index low. Since meropenem is a hydrophilic antibiotic, the conjugation of Meropenem to TP4 may increase the gross hydrophilicity of the peptide, thus improving the overall toxicity and antimicrobial profiles.^22^

### Conjugation Site Analysis

**Figure 4:**
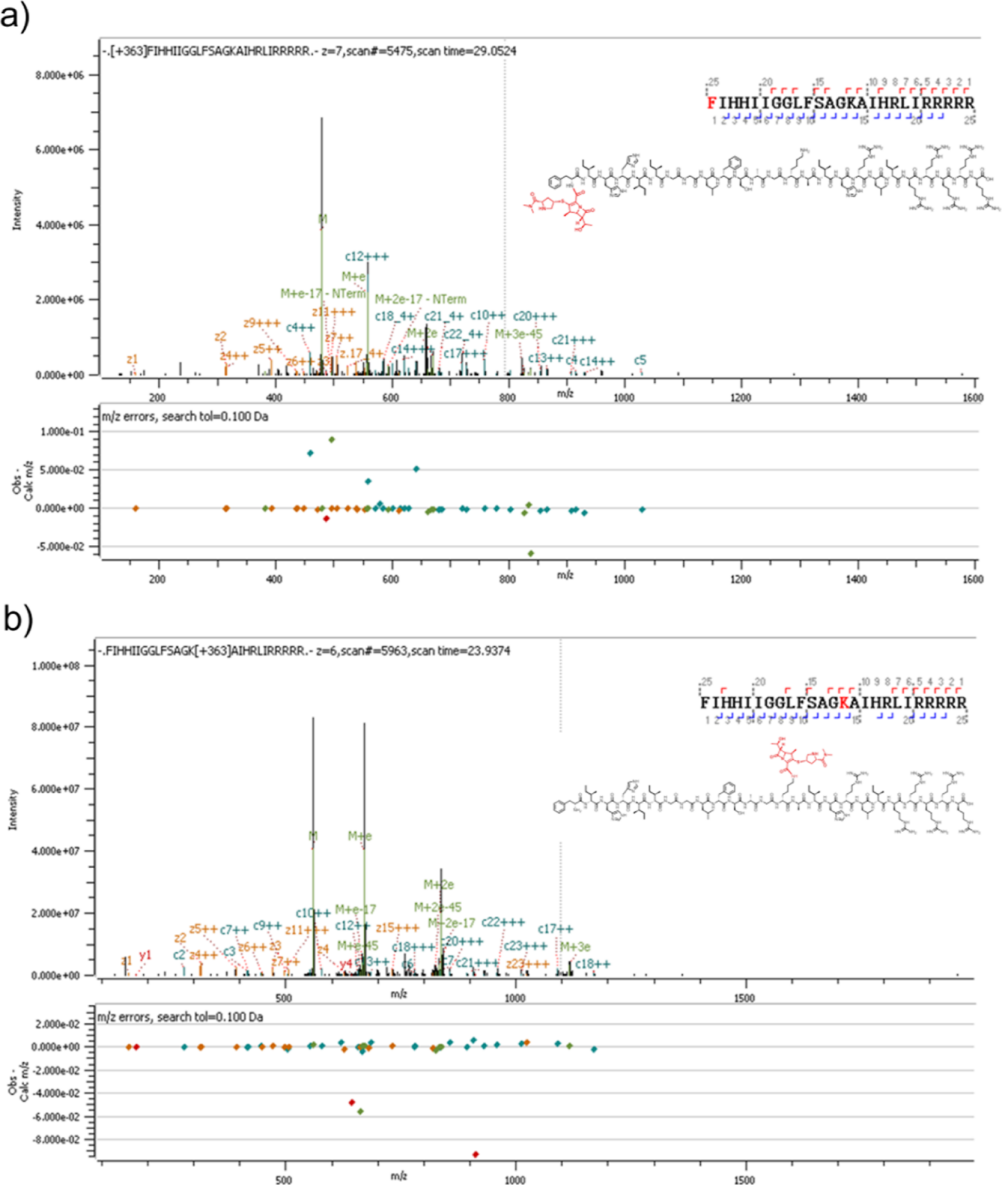
MS–MS fragmentation analysis of D-TP4-Meropenem conjugates. (a) TP4-N-Mero; (b) TP4-K-Mero.

To examine the conjugates at a structural level, the meropenem conjugation sites were analyzed by MS–MS fragmentation analysis. In line with the fact that unprotected TP4 has two major primary amine groups, the fragmentation analysis revealed that Meropenem was conjugated to the N-terminus (TP4-N-Mero) (Figure 4a) and to the internal lysine (TP4-K-Mero) (Figure 4b) residue of TP4.

### Purification and Antibacterial Activity of Isomers

The isomer with meropenem attached to the N-terminus (TP4-N-Mero) and that with meropenem attached to the internal lysine residue (TP4-K-Mero) were further purified from the mixture by using a reversed-phase C18 column; the mass spectrometry analysis of each purified conjugate can be seen in Supporting Information Figure S2. The spectra show identical *m*/*z* values and similar intensities for both isomers, indicating that the two forms have comparable stability during the process. TP4-N-Mero and TP4-K-Mero were then tested for their individual antibacterial activities against an NDM-1-positive MDR *E. coli* strain. As observed for the mixture, the individual conjugates retained similar activities to that of native L-TP4; meanwhile, meropenem showed no activity up to 64 μM. The minimum bactericidal concentration (MBC) of L-TP4 and both conjugates were comparable at about 8 μM against the NDM-1-positive strain (Table 2).

**Table 2:** Anti-NDM–*E. coli* Activity of D-TP4-meropenem Isomers^a^

AMP/conjugate	NDM *E. coli* BAA 2452	
	MIC	MBC
L-TP4	4–8	4–8
TP4-N-Mero	4–8	8
TP4-K-Mero	8	8
Meropenem	>64	>64

a Values are expressed in (μM).

### Hemolytic Properties of the Isomers

**Figure 5:**
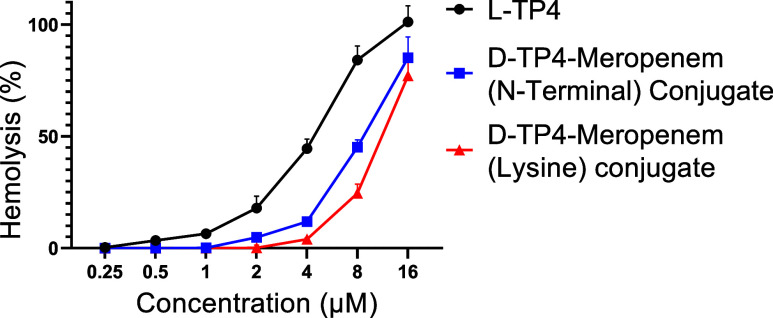
Hemolytic activity of TP4-meropenem isomers.

The hemolysis activities for both conjugates were lower than those of the parent TP4 peptide (Figure 5). Specifically, the hemolysis effects of L-TP4, TP4-N-Mero, and TP4-K-Mero were, respectively, observed to be 44.4 ± 4.3%, 11.8 ± 1.7%, and 3.9 ± 0.6% of the total cells at 4 μM; at 8 μM, the respective hemolysis proportions were 84.1 ± 6.2%, 45.1 ± 3.3%, and 24.4 ± 4.1%. Both L-TP4 and D-TP4 showed similar hemolytic potential (Supporting Information Figure S3). Based on these results, we concluded that conjugation with Meropenem improves the therapeutic profile of TP4, and TP4-K-Mero outperforms TP4-N-Mero in terms of hemolysis.

## Molecular Modeling Studies

### Membrane Interaction Potential

**Figure 6:**
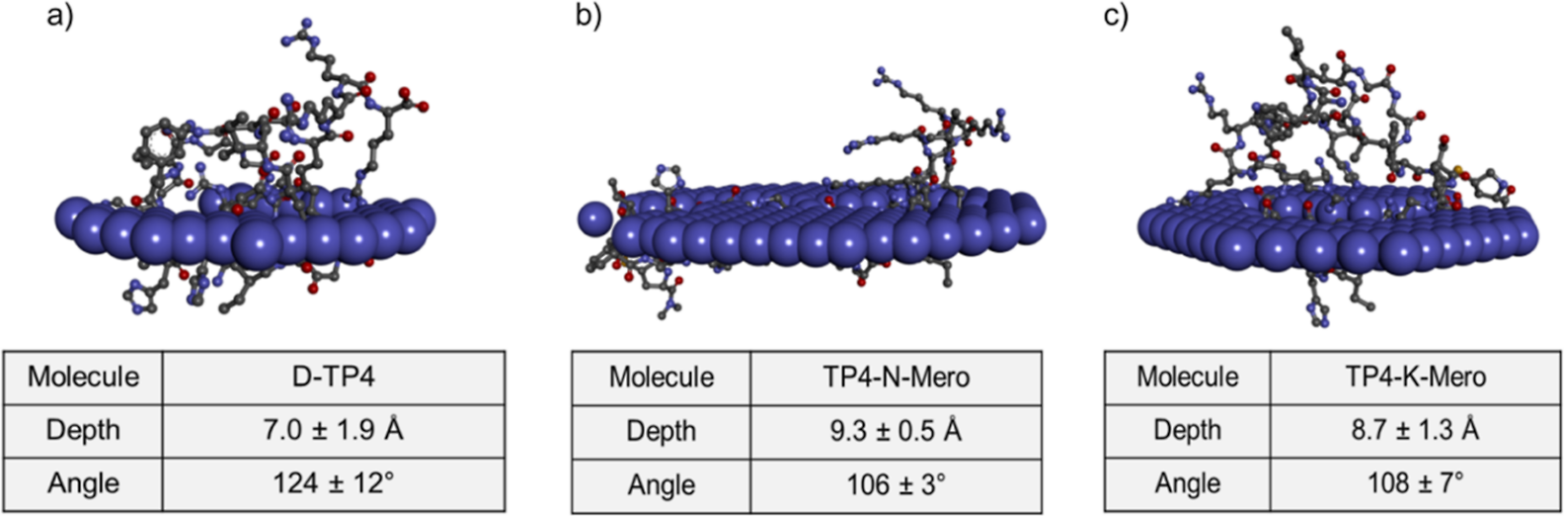
Position of protein on Gram-negative membrane (PPM) analysis. Proposed model for (a) D-TP4, (b) TP4-N-Mero, and (c) TP4-K-Mero.

Next, an *in silico* analysis was performed to better understand the physicochemical and interaction properties of the AMP-antibiotic conjugates. The conjugates were converted to 3D structures (Supporting Information Figure S4) and subjected to modeling studies. The interaction profiles of TP4 or the conjugates with Gram-negative cell membranes were simulated in a protein position in flat or curved membrane (PPM) analysis. The angles of interaction and depths of penetration were estimated. Compared with the results from TP4, the angles of interaction were slightly lower (Figure S3) and the depths were relatively higher (Figure 6) for the conjugates.

### LPS Binding Analysis

**Figure 7:**
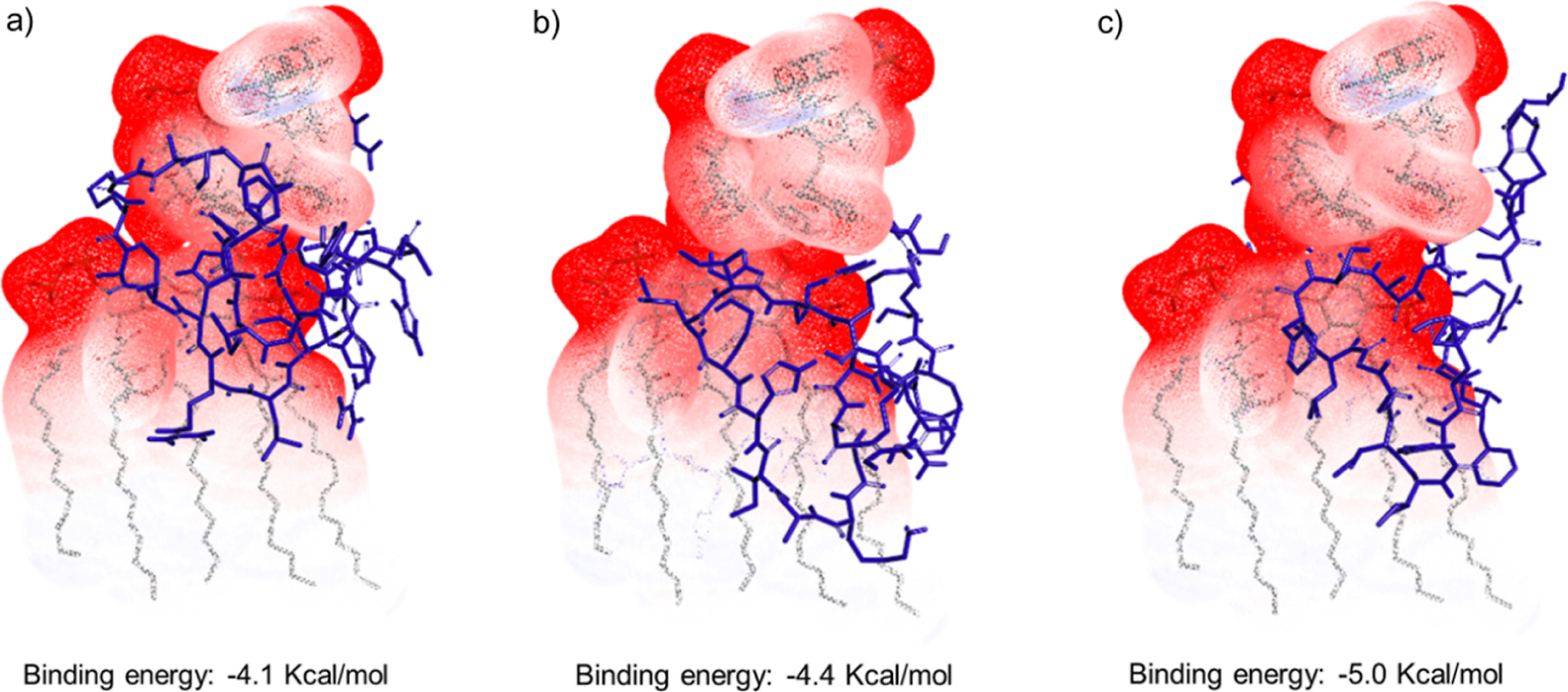
LPS binding analysis. Proposed model for (a) D-TP4, (b) TP4-N-Mero, and (c) TP4-K-Mero.

LPS are a crucial cell membrane component of Gram-negative bacteria. Interaction with the Gram-negative membrane composed of LPS is observed to be the primary target of the AMPs. To identify the LPS interaction of the conjugates, molecular docking analysis was performed. Significant binding energies with LPS were observed from the molecular docking analysis (Figure 7). This result is in line with the data obtained in the *in vitro* antibacterial study. Further, LPS binding has also been shown to exhibit neutralizing property leading to anti-inflammatory effects.^23^

### NDM-1 Inhibiting Potential

**Figure 8:**
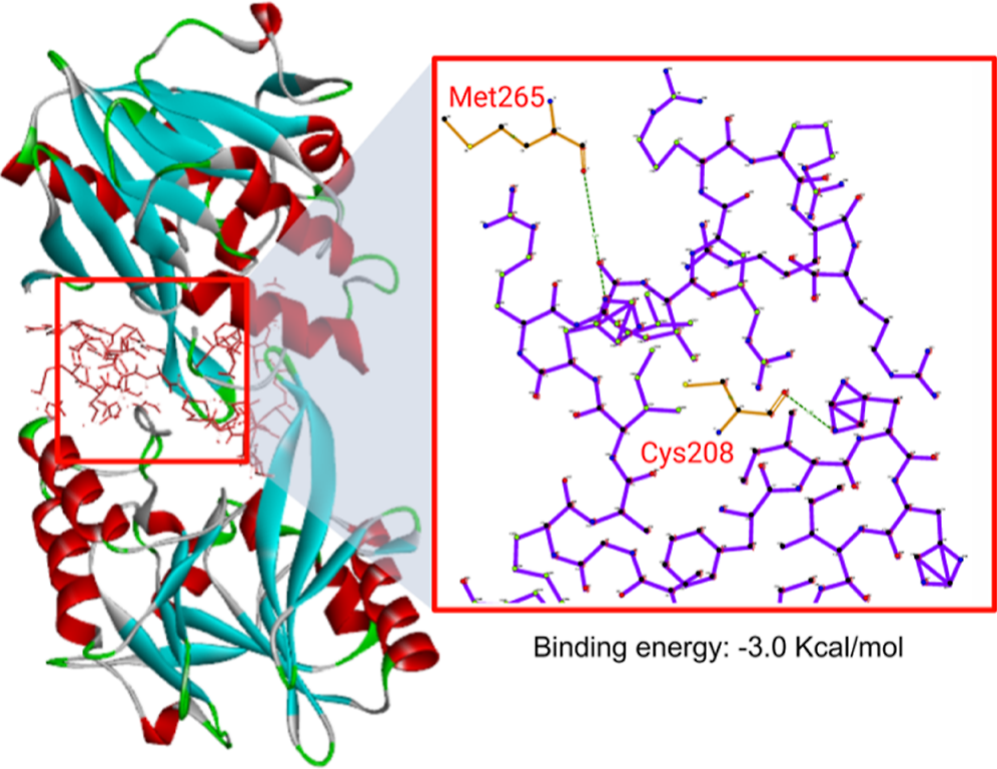
Proposed 3D model for the TP4-K-Mero interaction with NDM-1.

β-Lactamases produced by AMR pathogens act by hydrolyzing β-lactam rings of antibiotics, rendering the molecules inactive. As such, β-lactamase inhibitors have been developed to inhibit these enzymes in drug-resistant pathogens. Such molecules are often used in combination with first-line antibiotics. For instance, clavulanic acid, sulbactam, tazobactam, avibactam, and relebactam are often used in combination with common antibiotics, such as amoxicillin, piperacillin, or ceftazidime when targeting MDR pathogens.^24−26^ Encouragingly, growing evidence suggests that AMPs may also be useful as adjuvants or β-lactamase inhibitors, which can potentially exert synergistic antimicrobial activities with antibiotics.^27−29^ In light of these studies, our molecular docking results suggest that the conjugates may have β-lactamase-inhibiting effects due to their high binding affinity to the active site of the NDM-1 enzyme and H-bond formation on Cys208 and Met265. Cys208 is a conserved active residue site essential for Zn^2+^ binding (Figure 8). This residue is crucial for enzyme catalytic activity and stability.^30^ Thus, the conjugate developed can also be utilized in combination with other antibiotics to combat recalcitrant infections.

## Conclusions

TP4-Meropenem conjugates were synthesized, characterized, and examined for its therapeutic properties. The conjugates showed an antimicrobial activity similar to that of TP4, while the cytotoxicity to mammalian RAW264.7 and CCD-966SK cells was reduced dramatically. MS–MS fragmentation analysis showed that the conjugation of unprotected TP4 with Meropenem led to isomers at the N-terminus and the internal lysine residue of TP4. The resulting TP4-N-Mero and TP4-K-Mero conjugates showed an eminent antibacterial profile against NDM-1 *E. coli.* The conjugation also reduced the hemolytic effects of TP4, with TP4-K-Mero showing lower hemolysis activity than TP4-N-Mero. *In silico* analysis further revealed that the conjugates interact with the outer bacterial membrane and can bind to LPS and NDM-1. In conclusion, our AMP-antibiotic conjugates (together/separate) show an improved therapeutic profile compared to TP4 and Meropenem alone, especially toward MDR strains. Furthermore, the conjugates with the NDM-1 inhibiting effect may serve as a β-lactam inhibitor to resensitize antibiotics against drug-resistant pathogens.

## Materials and Methods

### Synthesis of TP4-Meropenem Conjugates and Characterization

Conjugation of Meropenem to D-TP4 was performed as described in a previous report with minor modifications.^31^ First, 10 mg of Meropenem sulfate was dissolved in 0.01 M 2-(N-morpholino) ethanesulfonic acid (MES) buffer at pH 7. In addition, 5 mg/mL TP4, 4 mg/mL *N*-ethyl-*N*′-(3-(dimethylamino)propyl) carbodiimide hydrochloride (EDC.HCl), and 2.5 mg/mL hydroxybenzotriazole (HOBt) were prepared in dimethyl sulfoxide (DMSO). Then, 100 μL of Meropenem, 100 μL of HOBt, and 100 μL of EDC.HCl were mixed in a glass vial. After a 30 min incubation with stirring, 100 μL of D-TP4 was added dropwise to the mixture, and the mixture was further stirred at room temperature for 16 h. The conjugates were purified using reversed phase-HPLC (Agilent) on a C4 column (Grace, Vydac, 5 μM) using ACN/H_2_O with 0.1% FA as the mobile phase. HPLC-MS (Waters Alliance 2695 HPLC module with a Xevo TQ-S micro triple quadrupole mass spectrometry) and HR-MS analysis using an LTQ Orbitrap XL ETD mass spectrometer (Thermo Fisher Scientific, San Jose, CA, USA) equipped with a standard ESI ion source were carried out to verify the molecular weights of the conjugates. MS–MS fragmentation analysis was performed to identify the conjugation site of Meropenem on TP4 using LC-ESI-MS on an Orbitrap fusion mass spectrometer (Thermo Fisher Scientific, San Jose, CA) and an EASY spray source (Thermo Fisher Scientific, San Jose, CA, USA). N-terminal and Lysine-conjugated Meropenem-TP4 molecules were purified using a C18 column (4.6 × 250 mm, 5 μM, C18 Luna, Phenomenex).

### Determination of MIC and MBC

*E. coli* 8–8, *A. baumannii* 2982, and *S. aureus* 13–1 were procured from the Bioresource Collection and Research Center (BCRC), Taiwan. NDM-1-positive *E. coli* BAA 2452 was procured from ATCC. All strains were cultured in Mueller Hinton broth (MHB). The concentration of bacteria was identified by the absorbance at OD_600_. The colony forming units (CFU) corresponding to different OD_600_ values were identified by plating on the Mueller Hinton agar (MHA) plate.

Antibacterial analysis was performed according to a standard protocol.^32^ Overnight cultures of test bacteria were prepared and freshly subcultured. Briefly, 50 μL of 1 × 10^6^ CFU/mL bacterial cells was treated with 50 μL of peptide or conjugate at different concentrations in a 96-well plate. The plates were incubated at 37 °C for 16–20 h. The lowest concentration of peptide/conjugate showing no growth was considered the minimum inhibitory concentration (MIC). To identify the MIC (MBC), 5 μL of incubated suspensions from growth-free wells was plated onto MHA and incubated overnight at 37 °C. The lowest concentration of peptide/conjugate showing no growth on the MHA plate was considered to be the MBC. The experiments were performed in three independent replicates, each in triplicate.

### Cytotoxicity and LDH Leakage Assay

RAW 264.7 murine macrophage cells, CCD966-SK human fibroblast cells, and HaCaT human keratinocytes were procured from BCRC, Taiwan. RAW264.7 and HaCaT cells were cultivated in DMEM supplemented with 10% FBS, streptomycin (100 μg/mL), and penicillin (100 U/mL) at 37 °C and 5% CO_2_. CCD966-SK were cultivated in MEM with 0.1 mM nonessential amino acids with 10% FBS, streptomycin (100 μg/mL), and penicillin (100 U/mL) at 37 °C and 5% CO_2_.

*In vitro* cytotoxicity of the TP4-Meropenem conjugates was evaluated with an alamarBlue assay. Briefly, 5 × 10^4^ RAW264.7 cells/well or 5 × 10^3^ CCD966-SK or 1 × 10^4^ HaCaT cells/well were seeded in 96-well plates for 24 h and then treated with different concentrations of peptide/conjugate. Controls included 0.1% Triton-X-treated cells and cells in a culture medium. After 24 h of treatment, the cells were washed and incubated with 90 μL of DMEM and 10 μL of alamarBlue reagent for 2 h. The absorbances at 570 and 600 nm were measured using a Spectra Max i3 (Molecular Devices, CA). Cell viability was calculated using the formula as described in the protocol. The LDH assay was carried out using the cytotoxicity detection kit PLUS according to a previously described protocol.^33^

### Hemolysis Analysis

Human RBCs were used to examine the hemolytic profiles of the peptides and conjugates. The RBCs were washed three times with PBS and centrifuged at 1000*g*. Then, 50 μL of a 2% RBC suspension was added to 50 μL of peptide/conjugate solution at different concentrations. The mixture was incubated at 37 °C for 1 h. The incubated mixture was centrifuged, and hemolysis was assessed by measuring the absorbance of the supernatant at 540 nm.

### Ligand Preparation and Molecular Docking Analysis

Molecular docking was carried out by converting 2D structures of selected ligands into 3D (Chemsketch); energy minimization was performed with the YASARA.^34^ The crystal structures of LPS and NDM-1 were downloaded and extracted from the RCSB protein data bank (PDB ID: 1QFG and 3Q6X) (Supporting Information Figure S5). The protein preparation was carried out using PyMol software; unnecessary water molecules and heteroatoms were deleted, and the structures were further converted to pdb files. Ligands and proteins were converted to a pdbqt format using Autodock tools. Molecular docking was performed in PyRx software utilizing Auto Dock Vina as algorithm.^35,36^ The grid box was generated covering the LPS molecule and a 10 Å area covering the binding sites of the NDM-1 enzyme. The binding energies, 3D docking poses, and 2D interaction images were examined using BIOVIA Discovery Studio Visualizer and LigPlot.

### Membrane Penetration Potential

The positions of peptides in the outer membrane of Gram-negative bacteria and penetration potential were analyzed using the PPM 3.0 (https://opm.phar.umich.edu/ppm_server3_cgopm) web server. The PDB files of the peptides and conjugates were used as input files to calculate the angle of interaction and penetration depth.^37,38^

## Supporting Information Available

Cytotoxicity analysis of the L-TP4 and D-TP4; mass spectra of purified conjugates; hemolysis analysis of L-TP4 and D-TP4; 3D structures of ligands; and 3D structures of LPS and NDM-1 β-lactamase (PDF)
