# Supplementary material for: Rejuvenation of Meropenem by Conjugation with Tilapia Piscidin-4 Peptide Targeting NDM-1 Escherichia coli
Source: ACS Omega. 2024 Jun 28;9(27):29756–64. doi: 10.1021/acsomega.4c03352 (PMC11238198; doi:10.1021/acsomega.4c03352)
Supplement: Supplementary file 1 — ao4c03352_si_001.pdf [file ao4c03352_si_001.pdf]

Rejuvenation of Meropenem by conjugation with Tilapia piscidin-4 peptide targeting NDM-1 *Escherichia coli*

Sanjay Prasad Selvaraj[a,b], Kuan- Hung Lin[c], Wen-Chun Lin[d] Ming-Feng You[d] , Tsung-Lin Li[c,e]*, Jyh-Yih Chen[d,f]*

[a]*Molecular and Biological Agricultural Science Program, Taiwan International Graduate Program, Academia Sinica, Taipei, 11529, Taiwan*

[b]*Graduate Institute of Biotechnology, National Chung Hsing University, Taichung, 402, Taiwan*

[c]*Genomics Research Center, Academia Sinica, Taipei, 11529, Taiwan*

[d]*Marine Research Station, Institute of Cellular and Organismic Biology, Academia Sinica, 23-10 Dahuen Rd, Jiaushi, Ilan, 262, Taiwan*

[e]*Biotechnology Center, National Chung Hsing University, Taichung City, 402, Taiwan*

[f]*The iEGG and Animal Biotechnology Center and the Rong Hsing Research Center for Translational Medicine, National Chung Hsing University, Taichung, 402, Taiwan*

***Correspondence author's mailing address:** Marine Research Station, Institute of Cellular and Organismic Biology, Academia Sinica, 23-10 Dahuen Rd, Jiaushi, Ilan, 262, Taiwan, Tel.: +886-920802111, Fax: +886-398971035; E-mail: zoocjy@gate.sinica.edu.tw

Table of content

S. No	List of Figures	Content	Page no.
1	Supplementary Figure S1	Cytotoxicity analysis of the L-TP4 and D-TP4. Cytotoxicity analysis on RAW264.7 (a) and HaCaT cells, (b) by alamar blue assay.	2
2	Supplementary Figure S2	Mass spectra of purified conjugates a) TP4-N-Mero; b) TP4-K-Mero.	2
3	Supplementary Figure S3	Hemolysis analysis of L-TP4 and D-TP4	3
4	Supplementary Figure S4	3D structures of ligands. a) D-TP4, b) TP4-N-Mero, c) TP4-K-Mero.	3
5	Supplementary	3D structures of a) Lipopolysaccharides (LPS) b)	5

	Figure S5	NDM-1 β-lactamase.	

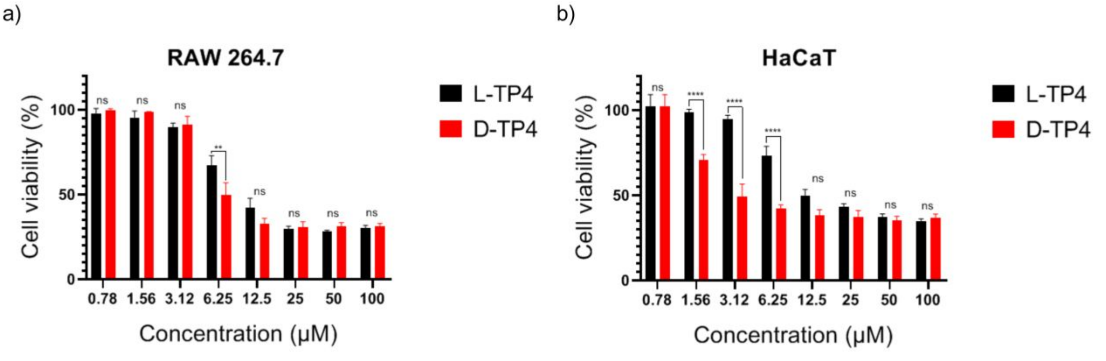

Supplementary Figure S1: Cytotoxicity analysis of the L-TP4 and D-TP4. Cytotoxicity analysis on RAW264.7 (a) and HaCaT cells, (b) by alamar blue assay. Significant differences compared to the control were identified by two-way ANOVA and Tukey's multiple comparison test (*$p < 0.05$; **$p < 0.01$; ***$p < 0.001$; ****$p < 0.0001$, ns – no statistical significance).

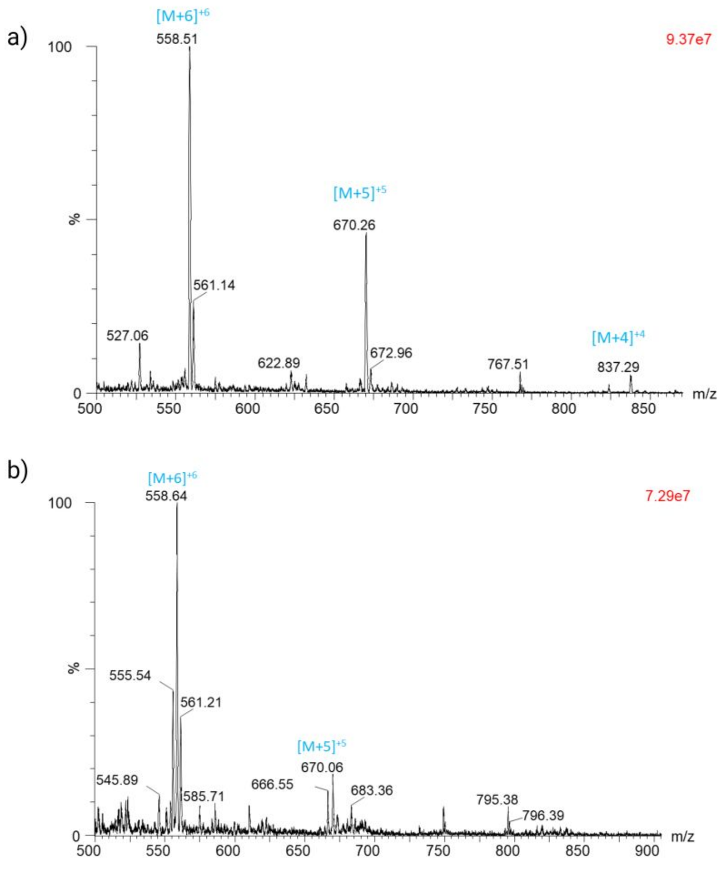

Supplementary Figure S2: Mass spectra of purified conjugates a) TP4-N-Mero; b) TP4-K-Mero.

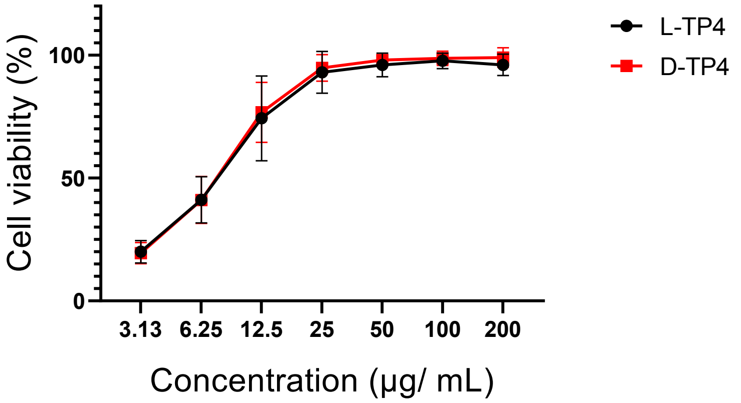

Supplementary Figure S3: Hemolysis analysis of L-TP4 and D-TP4.

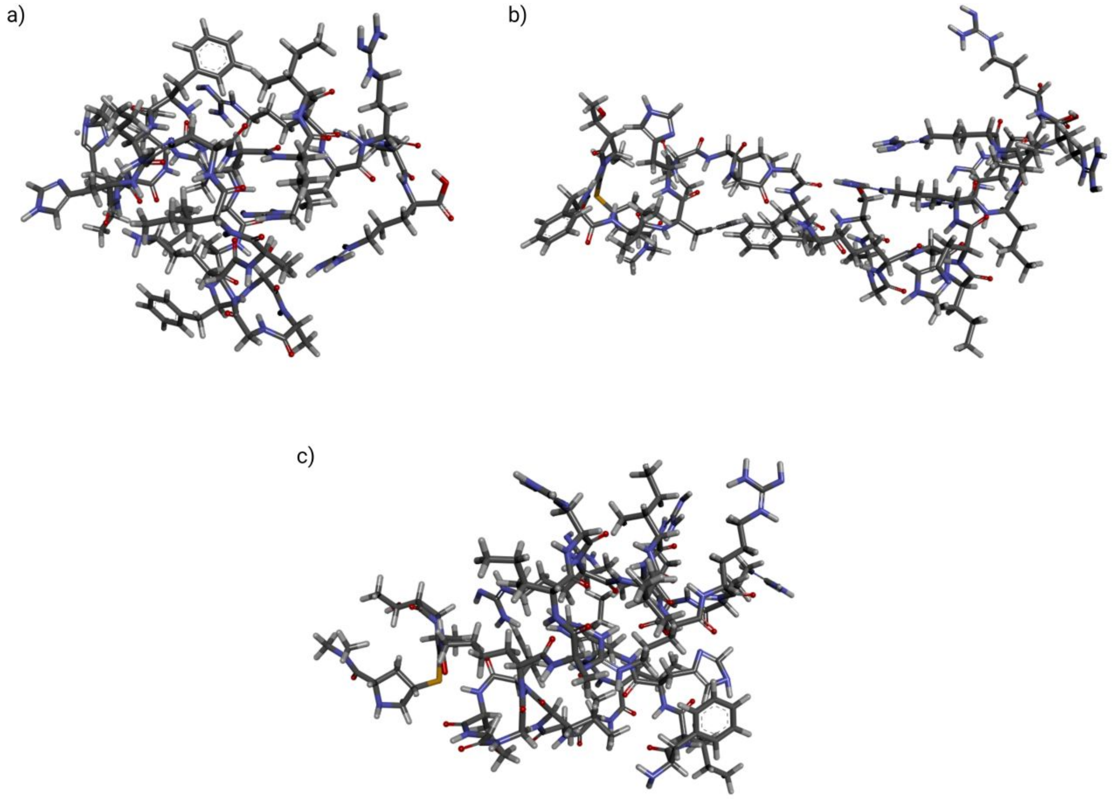

Supplementary Figure S4: 3D structures of ligands. a) D-TP4, b) TP4-N-Mero, c) TP4-K-Mero.

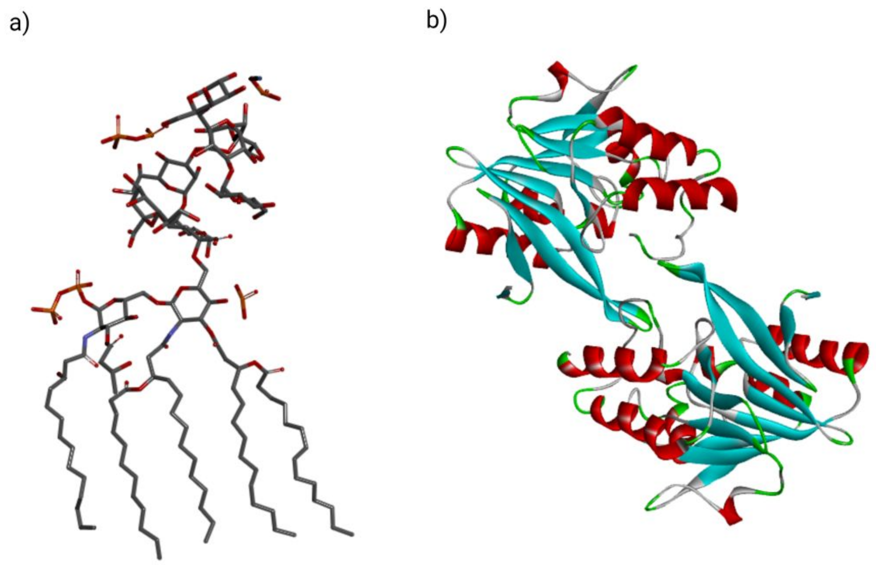

Supplementary Figure S5: 3D structures of a) Lipopolysaccharides (LPS) b) NDM-1 β-lactamase.
